# Cloning and Analysis of Gene Expression of Two Toll Receptors in Freshwater Pearl Mussel *Hyriopsis cumingii*

**DOI:** 10.3389/fphys.2018.00133

**Published:** 2018-03-05

**Authors:** Ying Huang, Keke Han, Qian Ren

**Affiliations:** ^1^Jiangsu Key Laboratory for Biodiversity and Biotechnology and Jiangsu Key Laboratory for Aquatic Crustacean Diseases, College of Life Sciences, Nanjing Normal University, Nanjing, China; ^2^College of Oceanography, Hohai University, Nanjing, China; ^3^Co-Innovation Center for Marine Bio-Industry Technology of Jiangsu Province, Lianyungang, China

**Keywords:** *Hyriopsis cumingii*, toll receptors, RNAi, innate immunity, anti-microbial peptides

## Abstract

Toll receptors are involved in innate immunity of invertebrates. In this study, we identify and characterize two Toll genes (named *HcToll4* and *HcToll5*) from triangle sail mussel *Hyriopsis cumingii. HcToll4* has complete cDNA sequence of 3,162 bp and encodes a protein of 909 amino acids. *HcToll5* cDNA is 4,501 bp in length and encodes a protein of 924 amino acids. Both deduced HcToll4 and HcToll5 protein contain signal peptide, extracellular leucine rich repeats (LRRs), and intracellular Toll/interleukin-1 (IL-1) receptor domains. Quantitative real-time PCR analysis revealed that *HcToll4* and *HcToll5* were largely distributed in the hepatopancreas and could be detected in the gills and mantle. *HcToll4* and *HcToll5* expression could respond to *Staphylococcus aureus, Vibrio parahaemolyticus*, White spot syndrome virus (WSSV) or Poly I:C challenge. RNA interference by siRNA results showed that *HcToll4* and *HcToll5* were involved in the regulation of *theromacin* (*HcThe*) and *whey acidic protein* (*HcWAP*) expression. Based on these results, *HcToll4* and *HcToll5* might play pivotal function in *H. cumingii* innate immune response.

## Introduction

Innate immune system is the first line of host defense against inevitably encounter pathogens and is conserved throughout evolution. The activation of innate immune response requires the recognition of pathogen-associated molecular patterns (PAMPs) by pathogen-recognition receptors (PRRs), such as Tolls/Toll-like receptors (TLRs), Nod-like receptors (NLRs), and RIG-like receptors (RLRs) (Bilak et al., 2003; Carty and Bowie, 2010). These PRRs can recognize the unique patterns of PAMPs as conserved molecular motifs on the surface of invading microbes (Imler and Zheng, 2004; Kawai and Akira, 2010) and subsequently trigger a series of rapid and effective immune responses, including the release of molecules and circulating humoral and cellular components (Medzhitov, 2007).

In *Drosophila melanogaster*, Toll pathways are the major regulators of the humoral and cellular immune responses, which control the expression of genes regulated by microbial infection (Hoffmann and Reichhart, 2002; Valanne et al., 2011). Gram-positive bacteria, fungi, or certain viruses activate the Toll pathway, thus leading to the systemic production of antimicrobial peptides (AMPs) that kill infective pathogens in *D. melanogaster* (Beutler, 2009; Hetru and Hoffmann, 2009). The key components of the Toll pathway in *Drosophila* include the cytokine-like ligand Spätzle, receptor Toll, intracellular adaptor myeloid-differentiation-factor-88 (MyD88), adaptor protein Tube, protein kinase Pelle, and nuclear factor-κB (NF-κB)-family transcription factors such as Dorsal and Dorsal-related immunity factor (Dif). After Toll pathway activation, Dorsal or Dif translocates into the nucleus, leading to the expression of several *AMP* genes (Belvin and Anderson, 1996; Lemaitre and Hoffmann, 2007; Valanne et al., 2011).

In vertebrates, TLRs directly recognize and bind the PAMPs of multifarious pathogens and act as a bridge between the innate and adaptive immunity (Akira et al., 2001). Vertebrate TLRs have extracellular leucine rich repeat (LRR) domains that mediate the recognition of PAMPs; transmembrane and intracellular Toll/interleukin-1 (IL-1) receptor (TIR) domains are required for downstream signal transduction (Bowie and O'Neill, 2000; Kawai and Akira, 2010). Since the identification of the first Toll protein in *D. melanogaster* (Belvin and Anderson, 1996), Tolls or TLRs have been identified in many vertebrates and invertebrates. Approximately 10 and 12 functional TLRs have been characterized in humans and mice, respectively; among these, TLR1–TLR9 are conserved in both species (Kawai and Akira, 2010). Approximately 222 and 72 TLR-related genes existed in sea urchin and amphioxus genomes, respectively (Satake and Sekiguchi, 2012). Seventeen different kinds of TLRs have also been found in fish (Palti, 2011), and 16 TLRs have been described in lamprey genome (Armant and Fenton, 2002). In mollusks, Toll genes have been isolated in some mussels, including Zhikong scallop *Chlamys farreri* (Qiu et al., 2007), mollusk oyster *Crassostrea gigas* (Zhang et al., 2011), noble scallop *Chlamys nobilis* (Lu et al., 2016), triangle-shell pearl mussel *Hyriopsis cumingii* (Ren et al., 2014), and mediterranean mussel *Mytilus galloprovincialis* (Toubiana et al., 2013). Mollusca Toll receptors have an extensive and constitutive tissue-level expression and are closely involved in innate immune responses against pathogenic infection (e.g., bacteria, virus; Qiu et al., 2007; Zhang et al., 2011; Toubiana et al., 2013; Ren et al., 2014; Lu et al., 2016).

This study aims to (1) identify new Toll genes in *H. cumingii*, (2) clone their complete nucleotide sequences, (3) investigate their phylogenic relationships, (4) measure their constitutive tissue-specific expression, (5) quantify their expression level following *in vivo* challenges with bacteria or virus, and (6) investigate their involvement in innate immune response by exploring their possible relationship with downstream immune related genes.

## Materials and methods

### Triangle-shell pearl mussels and immune challenge

Two hundred *H. cumingii* specimens (15 g mean weight) were obtained from Wuhu City, Anhui Province, China and were cultured in oxygenated freshwater at 25°C. Different tissues, including hemocytes, hepatopancreas, gills, and mantle from five normal mussels were collected, pooled, and then immediately frozen in liquid nitrogen and stored at −80°C until total RNA extraction. In the experimental groups, approximately 100 μl of *Staphylococcus aureus* (3 × 10^7^ cells), *Vibrio parahaemolyticus* (3 × 10^7^ cells), White spot syndrome virus (WSSV) (10^5^ copies/ml), or polyinosinic-polycytidylic acid poly I:C (0.5 μg/μl) were injected into the adductor muscles of *H. cumingii* (each group contained 20 mussels) using a 1 ml syringe. The hepatopancreas and gills of five mussels were collected after 2, 6, 12, and 24 h for RNA isolation.

### Molecular cloning and sequencing

Total RNA was extracted from various samples using RNApure High-purity Total RNA Rapid Extraction Kit (Spin-column; Bioteke, Beijing, China). Two expressed sequence tag sequences similar to the Toll genes were identified after analyzing the transcriptome data of the hepatopancreas from *H. cumingii* (data unpublished) and were used to clone the full length of Toll cDNAs by Rapid Amplification of cDNA Ends (RACE) method using gene-specific primers (Table 1). The 5′- and 3′-RACE were performed on RNA extracted from hepatopancreas using the Clontech SMARTer™ RACE cDNA Amplification Kit (Takara, Dalian, China) and an Advantage 2 cDNA Polymerase Mix (Clontech) according to the manufacturer's instructions. The PCR products were ligated into the pEASY-T3 vector (TransGen Biotech, Beijing, China), transformed into competent *Escherichia coli* DH5α cells, plated onto LB–agar Petri dishes, and incubated overnight at 37°C. Positive clones containing the insert with the expected size were identified by colony PCR, and the selected clones were sequenced.

**Table 1:** Sequences of the primers used in the study.

**Primers name**	**Sequences (5′-3′)**
HcToll4-F	CCATTCTCAATCACAAGCGAGGCACAG
HcToll4-R	ACAACCGTCTTTCTGCTGCGATGGATG
HcToll5-F	ATCGGTGTAGCAGCAACTCAACCTCAT
HcToll5-R	TGACGAAAACTGTCCCTCCTTTGCTTG
**UPM**	
Long	CTAATACGACTCACTATAGGGCAAGCAGTGGTATCAACGCAGAGT
Short	CTAATACGACTCACTATAGGGC
5′-CDS Primer A	T_25_VN
SMARTer II A oligo	AAGCAGTGGTATCAACGCAGAGTACXXXXX
3′-CDS primer A	AAGCAGTGGTATCAACGCAGAGTAC(T)30VN
HcToll4-RT-F	ATGGAAAGGACGAGAACA
HcToll4-RT-R	GGGTGGGACAATAATAGGA
HcToll5-RT-F	TCGTCAAGTCATAGAGCAGA
HcToll5-RT-R	GAAAGGAGAAACCAACAGAA
HcWAP-RT-F	TGTAATGTTGACGGGAGTG
HcWAP-RT-R	CTGTTTTGTTTTGATGGCT
HcThe-RT-F	CACAGTTGGTGGTTCAGTAA
HcThe-RT-R	CGAATCCTTCAGTAGATGGT
Hcactin-RT-F	GTGGCTACTCCTTCACAACC
Hcactin-RT-R	GAAGCTAGGCTGGAACAAGG

*X = undisclosed base in the proprietary SMARTer oligo sequence. N = A, C, G, or T; V = A, G, or C*.

### Bioinformatics analysis

cDNA and amino acid sequence similarity searches were performed using BLAST algorithm (http://blast.ncbi.nlm.nih.gov/Blast.cgi). Protein prediction was performed using software at the ExPASy (http://web.expasy.org/). Signal peptide and domain organization were predicted with SMART (http://smart.embl-heidelberg.de/). The calculated molecular weight and predicted isoelectric point were determined by ExPASy (http://web.expasy.org/compute_pi/). Protein multiple alignment analyses were performed using MEGA 5.05 and GENDOC software. The phylogenetic relationship of HcToll4 and HcToll5 was analyzed using the neighbor-joining (NJ) method with the MEGA 5.05 program and bootstrapping of 1,000 replicates.

### Gene expression analysis by qRT-PCR

Total RNA was extracted from the aforementioned samples. A total of 500 ng RNA from each sample was reverse-transcribed with PrimeScript® First Strand cDNA Synthesis Kit (Takara, Dalian, China) with the Oligo-d(T) Primer according to the protocol of the manufacturer. The synthesized cDNA samples were stored at −20°C prior to use in qRT-PCR assays. Primers were designed using the Primer Premier 5 software and are listed in Table 1. Melting curve was analyzed to select the optimum primer pairs. qRT-PCR was performed in a 10.0 μl of reaction mix containing 1.0 μl of cDNA sample, 5.0 μl of 2 × SYBR® *Premix Ex Taq* II (Tli RNaseH Plus) (Takara, Dalian, China), 0.2 μl of each primer (10 μM), and 3.6 μl of nuclease-free water. The qRT-PCR cycling conditions were as follows: 95°C for 30 s, 40 cycles of 95°C for 5 s, and 60°C for 30 s. Finally, melting curve analysis was conducted. The expression of β-actin in tissues and cells is relatively constant, and it is highly conserved among different species, so it was adopted as a housekeeping gene for internal standardization. A negative control without any cDNA template was included in the qRT-PCR analysis. The sequences of *HcToll4* and *HcToll5* specific primers and β-actin primers used in qRT-PCR are shown in Table 1. All samples were analyzed in triplicate during the qRT-PCR analysis. The expression level of *HcToll4* or *HcToll5* in different tissues was calculated using the comparative CT method, in which the discrepancy (ΔCT) between the CT of the *HcToll4* or *HcToll5* and β-actin was firstly calculated. Then the expression level of *HcToll4* or *HcToll5* was calculated by 2^−ΔCT^. The relative expression levels after immune challenge were calculated using 2^−ΔΔCT^ method (Livak and Schmittgen, 2001). Data from the qRT-PCR experiments were expressed as the means ± SE.

### Synthesis of siRNAs, RNAi assay, and detection of mRNA

Based on the sequences of *HcToll4* and *HcToll5*, small interfering RNA (siRNA) was synthesized *in vitro* with a commercial kit according to the manufacturer's instructions (Takara, Japan). The siRNAs used were *HcToll4*-siRNA (5′-GGUGGAGACUGAAGGACUU-3′), *HcToll5*-siRNA (5′-GCAAGAAGCUCGGAUGCAA-3′), and the sequence of siRNA was scrambled to generate the control *HcToll4*-siRNA-scrambled (5′-UGAGGGAAGCUGUAGCUGA-3′), *HcToll5*-siRNA-scrambled (5′-GACAUGAGAGCCGACAUAG−3′).

The RNA interference (RNAi) assay was conducted in mussels by injecting siRNA (40 μg) into each mussel using a 1 ml sterile syringe. In brief, 20 μg of siRNA and *V. parahaemolyticus* (3 × 10^7^ cells) were co-injected into the mussel at a volume of 100 μl per mussel. At 16 h after the co-injection, 20 μg of siRNA (100 μl/mussel) was injected into the same mussel. Simultaneously, the siRNAs-scrambled (20 μg; 100 μl/mussel) and *V. parahaemolyticus* (3 × 10^7^ cells) were co-injected into the mussel. At 16 h after the co-injection, the siRNAs-scrambled (20 μg; 100 μl/mussel) were injected into the same mussel. *V. parahaemolyticus* (3 × 10^7^ cells; 100 μl/mussel) alone was included in the injections as a positive control.

After RNAi treatment, the expression of downstream immune effector genes, *theromacin* (*HcThe*) and *whey acidic protein* (*HcWAP*) were detected by qRT-PCR with gene specific primers (Table 1). *Hcactin* was amplified as the control in the qRT-PCR assay. The assays were biologically repeated three times.

### Statistical analysis

The numerical data collected from three independent experiments were processed using one-way analysis of variance (ANOVA). Student's *t*-test was employed to test significant differences.

## Results

### Cloning of HcToll4 and HcToll5

**Figure 1:**
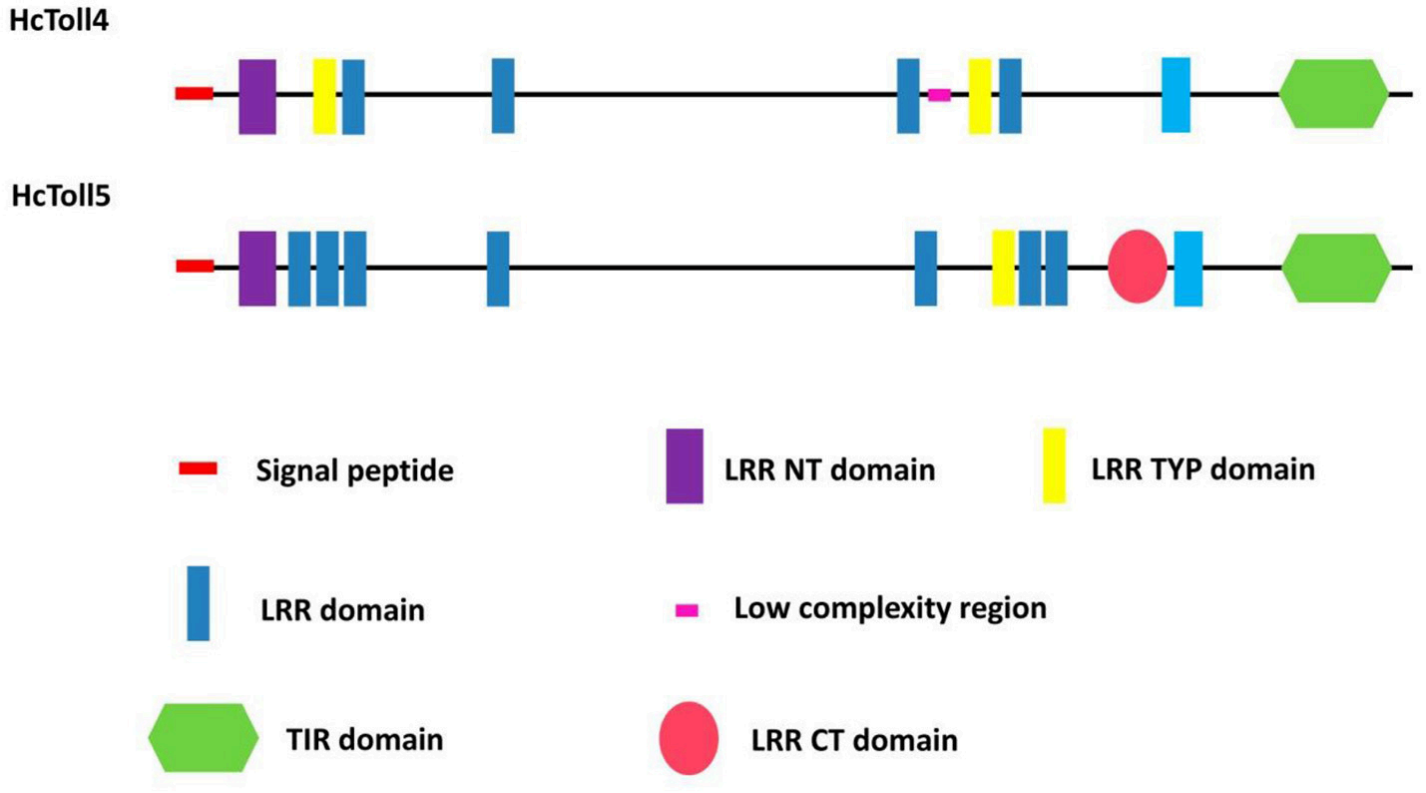
Domain organization of HcToll4 and HcToll5 from mussel.

A 3,162 bp nucleotide sequence representing the complete cDNA sequence of *HcToll4* (MG763893) was obtained (Figure S1A). The cDNA included a 111 bp 5′ untranslated region (UTR), a 3′ UTR of 321 bp with a poly (A) tail, and an open reading frame (ORF) of 2,730 bp. The ORF encoded a protein consisting of 909 amino acid residues with a predicted molecular weight of 104 kDa and a theoretical isoelectric point of 6.29. SMART analysis shows that HcToll4 contains a 17 amino acid (aa) signal peptide, a 37 aa Leucine rich repeat N-terminal (LRR_NT) domain, 2 24 aa LRRs, typical (most populated) subfamily (LRR_TYP) domains, 4 LRRs with aa length of 24–28, and a 141 aa intracellular Toll/interleukin-1 (IL-1) receptor (TIR) domain (Figure 1).

The full length *HcToll5* (MG763892) cDNA contains 4,501 nucleotides, including an 138 bp 5′UTR, a 2,775 bp ORF that encodes a 924 amino acid protein, and a 1,588 bp 3′ UTR with a poly (A) tail (Figure S1B). Similar to HcToll4 domain organization, HcToll5 contains a 18 aa signal peptide, a 37 aa LRR_NT domain, 7 LRR domains with aa length of 23–28, a 24 aa LRR_TYP domain, a Leucine rich repeat C-terminal (LRR_CT) domain of 57 aa, and a 139 aa TIR domain (Figure 1). The predicted HcToll5 protein has a molecular mass of 105.6 kDa and an isoelectric point of 6.12.

### Multiple sequence alignment and phylogenetic analysis

**Figure 2:**
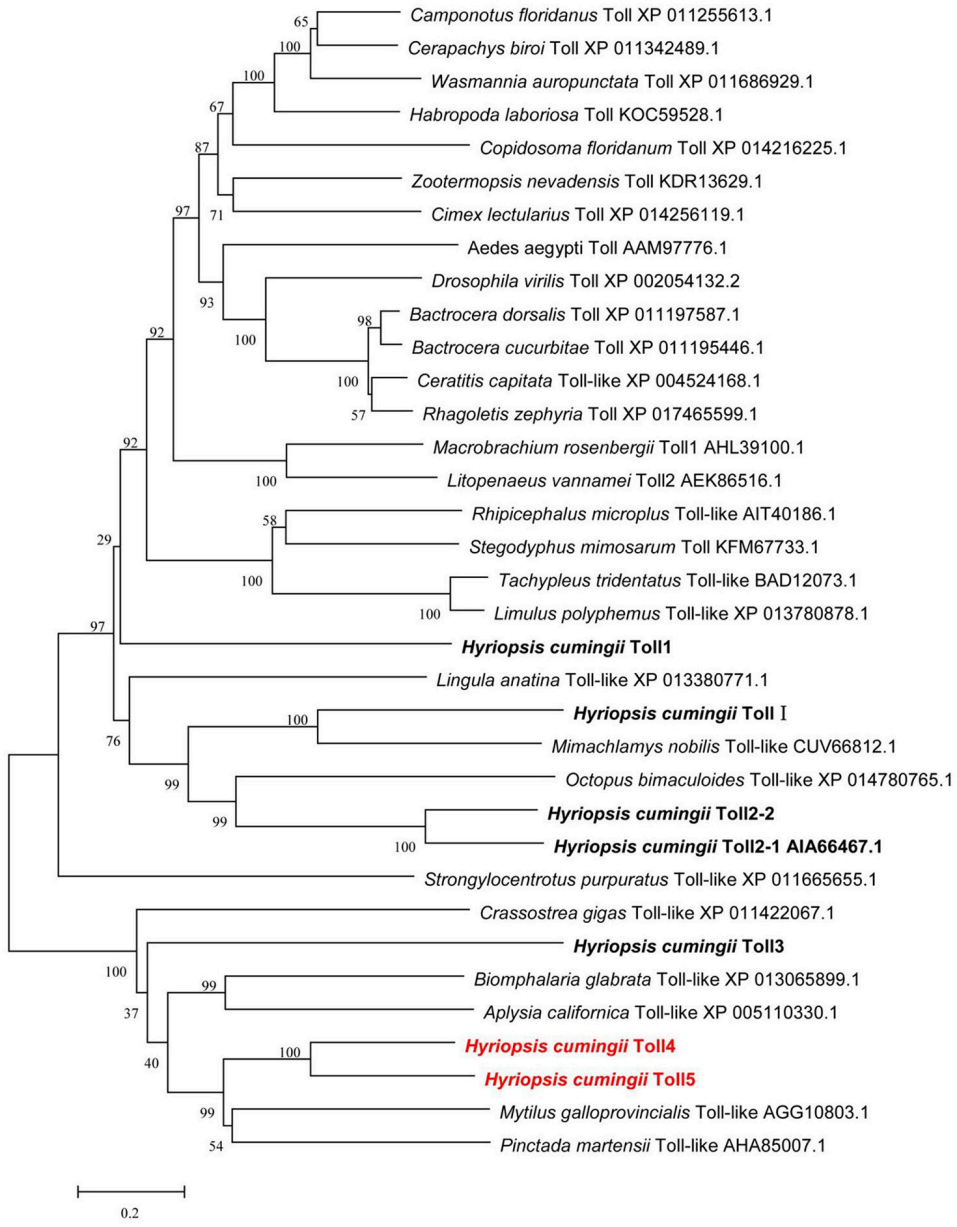
Phylogenetic analysis of HcToll4 and HcToll5 with other Toll receptors or TLRs. Phylogenetic tree was conducted with MEGA 5.05 using NJ method, and the reliability of the branching was tested by bootstrap resampling (1,000 pseudo-replicates).

Multiple amino acid sequence alignments showed that HcToll4 and HcToll5 were highly conserved with each other, especially in the TIR motif (Figure S2). BLASTX result showed that HcToll4 was 35% identical with Toll-like receptor from *M. galloprovincialis*, 33% with Toll-like receptor 4 from *Pinctada martensii*, 32% with Toll-like receptor 4 from *C. gigas*, and 30% with Toll-like receptor 4 from *Biomphalaria glabrata*. HcToll5 shared 34% identity with *C. gigas* Toll-like receptor 2, 33% identity with *M. galloprovincialis* Toll-like receptor, 30% with *P. martensii* Toll-like receptor 4 and *Aplysia californica* Toll-like receptor 3. The phylogenetic tree based on amino acid sequences from multiple Toll families was constructed by the NJ method with the assistance of MEGA software 5.05 (Figure 2). HcToll4 and HcToll5 were clustered with other bivalve Toll family members from *M. galloprovincialis* and *P. martensii* and then grouped with gastropoda Tolls from *B. glabrata* and *A. californica*.

### Tissue distributions of HcToll4 and HcToll5 mRNAs

**Figure 3:**
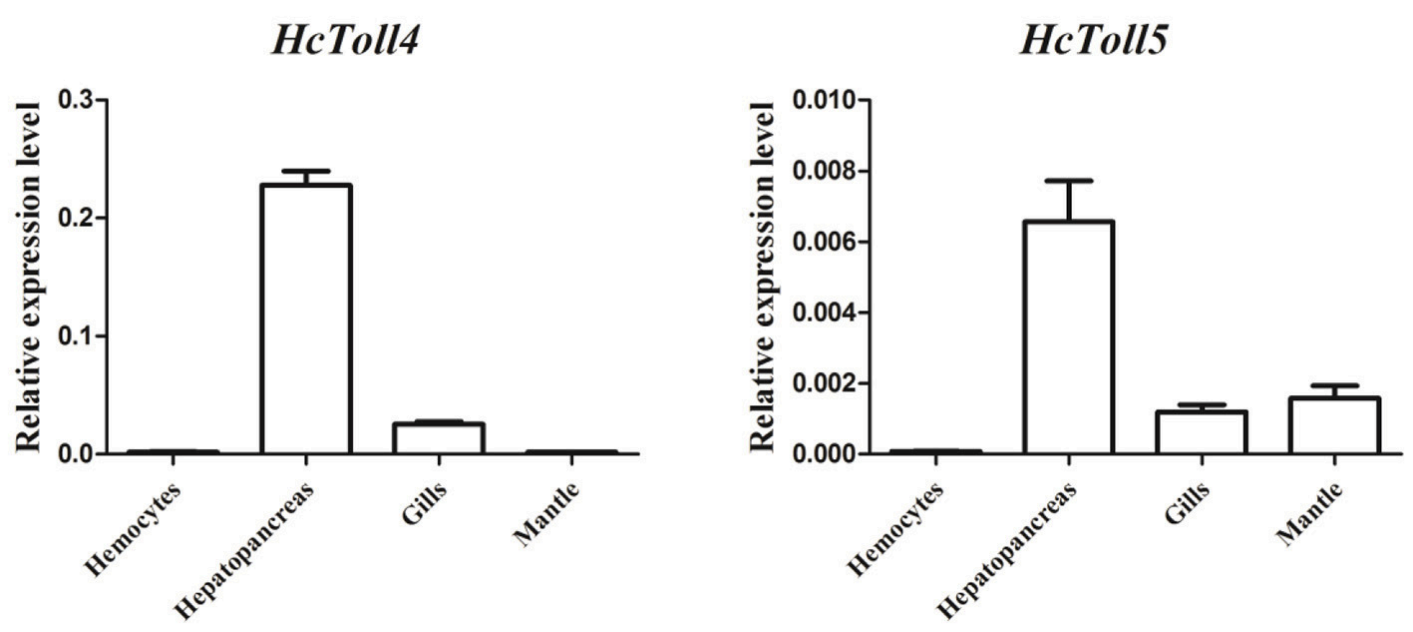
qRT-PCR analysis of *HcToll4* and *HcToll5* in the hemocytes, hepatopancreas, gills, and mantle of *H. cumingii*.

Quantitative RT-PCR analysis of various tissues including hemocytes, hepatopancreas, gills, and mantle showed the expression levels of *HcToll4* and *HcToll5* in *H. cumingii* (Figure 3). The highest *HcToll4* transcript was observed in hepatopancreas, followed by in gills, but was absent in hemocytes and mantles. *HcToll5* is largely distributed in hepatopancreas as compared in mantles and gills.

### Transcriptional regulation of HcToll4 after immune challenge

**Figure 4:**
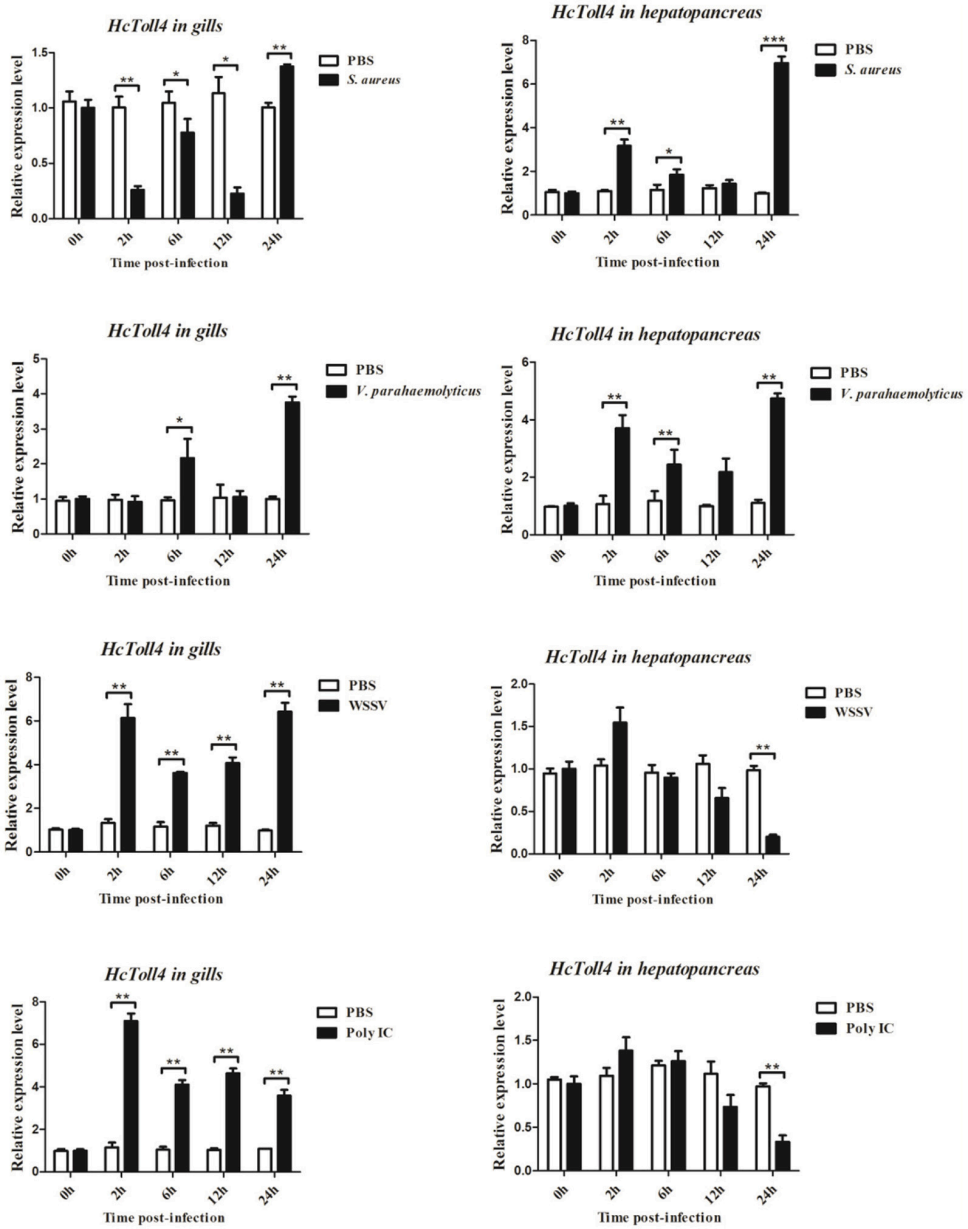
Analysis of *HcToll4* expressions in the gills and hepatopancreas from the mussels challenged with *S. aureus, V. parahaemolyticus*, WSSV, or Poly I:C using qRT-PCR methods. β-actin was used as a reference gene for internal standardization. Each bar represents the value from three independent PCR amplifications and quantifications. Statistical significance is indicated by asterisks (^*^P < 0.05, ^**^P < 0.01, ^***^P < 0.001) compared with that of the control.

The expression patterns of *HcToll4* in gills and hepatopancreas under conditions of *S. aureus, V. parahaemolyticus*, WSSV or poly I:C challenge were observed using qRT-PCR to investigate the roles of *HcToll4* on the anti-bacterial and anti-virus innate immunity of *H. cumingii* (Figure 4). After *S. aureus* stimulation, the mRNA expression level of *HcToll4* in gills was down-regulated at 2–12 h and then up-regulated at 24 h compared with that in the PBS control group, *HcToll4* in hepatopancreas was up-regulated at 2 h, slowly down-regulated from 6 to 12 h, and finally increased to the highest level at 24 h. After challenge with *V. parahaemolyticus, HcToll4* expression in gills was increased at 6 and 24 h, whereas that in hepatopancreas was significantly up-regulated at 2 and 24 h. After a significant increase at 2 h post WSSV stimulation, the mRNA expression of *HcToll4* in gills decreased at 6 h and then gradually increased from 12 to 24 h, whereas that in hepatopancreas was also increased at 2 h and then decreased from 6 to 24 h as compared with that in the PBS group. In poly I:C stimulated group, *HcToll4* in gills was quickly increased at 2 h and then slowly down-regulated from 6 to 24 h, whereas that in hepatopancreas showed no significant changes from 2 to 12 h and was down-regulated at 24 h.

### Time-course expression profiles of HcToll5 after immune challenge

**Figure 5:**
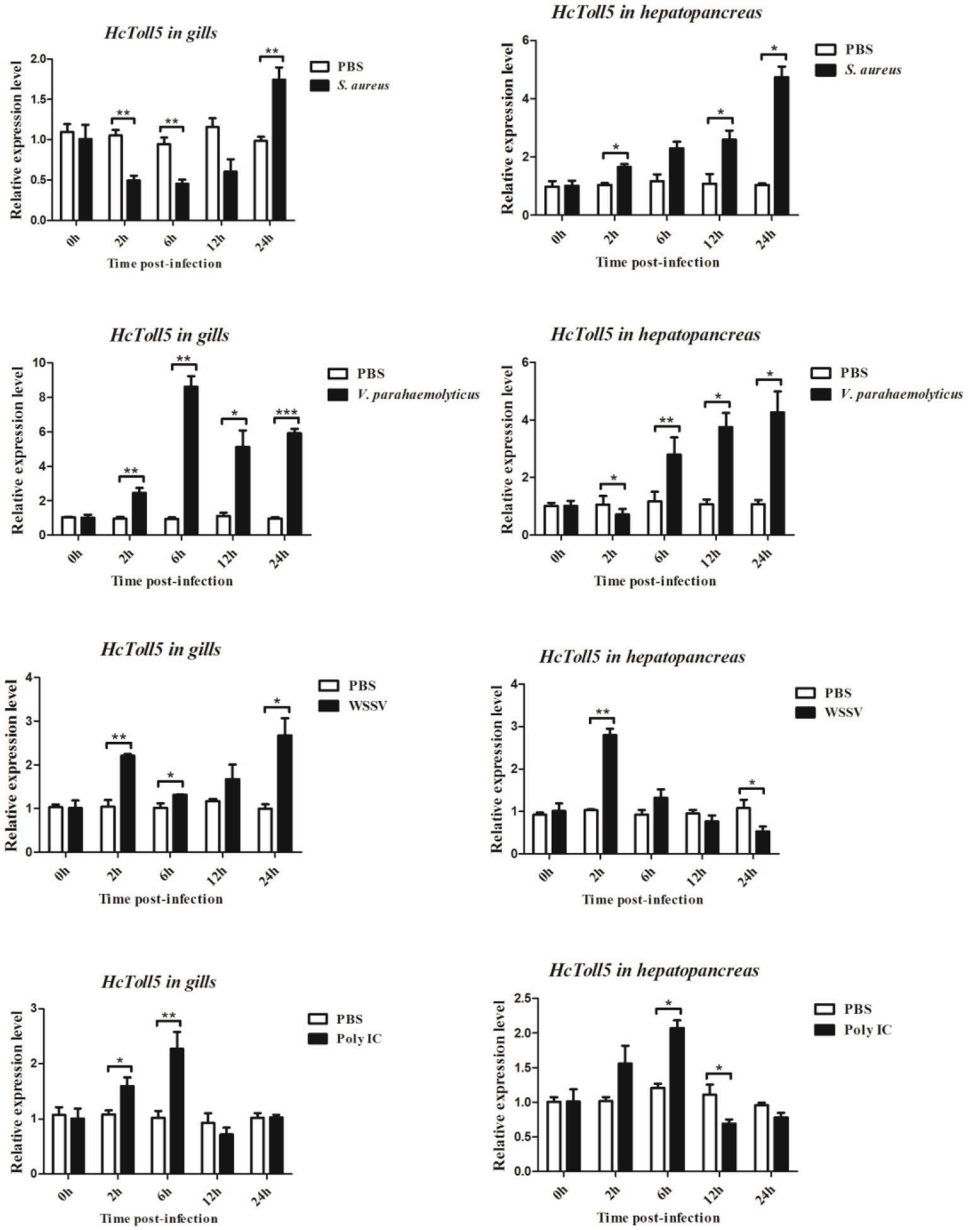
Analysis of *HcToll5* expressions in the gills and hepatopancreas from the mussels challenged with *S. aureus, V. parahaemolyticus*, WSSV, or Poly I:C using qRT-PCR methods. β-actin was used as a reference gene for internal standardization. Each bar represents the value from three independent PCR amplifications and quantifications. Statistical significance is indicated by asterisks (^*^P < 0.05, ^**^P < 0.01, ^***^P < 0.001) compared with that of the control.

The mRNA expression levels of *HcToll5* in gills and hepatopancreas were investigated from 2 to 24 h after four immune challenges (Figure 5). After *S. aureus* challenge, *HcToll5* mRNA in gills dropped to a low level from 2 to 12 h, then increased to its highest level at 24 h, whereas that in hepatopancreas was continuously up-regulated from 2 to 24 h. *HcToll5* expression levels in gills increased to the highest level by 6 h after *V. parahaemolyticus* challenge and then slowly decreased from 12 to 24 h; whereas that in hepatopancreas began to decrease within 2 h, increased from 6 to 24 h, and reached maximum at 24 h. In WSSV stimulated group, *HcToll5* mRNA in gills was increased at 2 h, and its expression level reached a second peak at 24 h; whereas that in hepatopancreas was significantly up-regulated at 2 h and then gradually decreased to the lowest level from 6 to 24 h. After poly I:C challenge, *HcToll5* expression quickly increased to the highest level at 6 h both in gills and hepatopancreas and then decreased from 12 to 24 h to the control level.

### RNAi of HcToll4 or HcToll5 suppressed the expression of AMPs

**Figure 6:**
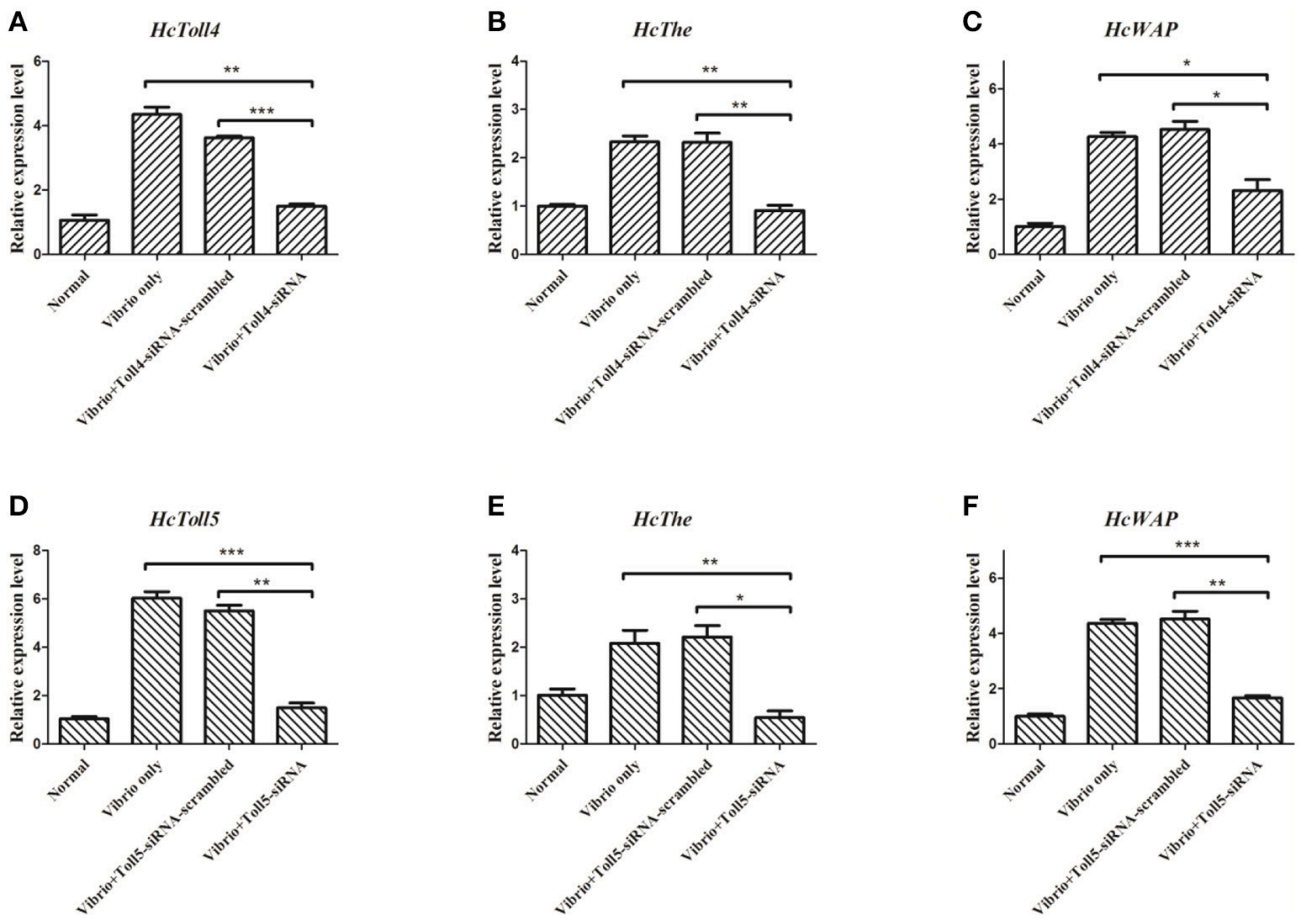
Knockdown of *HcToll4* or *HcToll5* and detection of *HcThe* and *HcWAP* expressions. qRT-PCR analysis of the RNA interference efficiency of *HcToll4* **(A)** or *HcToll5* **(D)** in gills of *H. cumingii*. The experiments were divided into four groups (normal group, 36 h *Vibrio* challenge group, *Vibrio* 36 h plus Toll-scrambled-siRNA group, and *Vibrio* 36 h plus Toll-siRNA group). Expression of *HcThe* mRNA in mussels after injection with *HcToll4*-siRNA **(B)** or *HcToll5*-siRNA **(E)** at 36 h. Expression of *HcWAP* in mussels after injection with *HcToll4*-siRNA **(C)** or *HcToll5*-siRNA **(F)** at 36 h. Five mussels were used in each group in this assay. Different asterisks indicate significant differences between groups, ^*^P < 0.05, ^**^P < 0.01, ^***^P < 0.001. The experiment was repeated three times.

Two sequences specific siRNAs (*HcToll4*-siRNA and *HcToll5*-siRNA) were respectively injected into mussels to silence the *HcToll4* or *HcToll5* expression and further reveal the role of *HcToll4* and *HcToll5 in vivo. HcToll4* or *HcToll5* mRNA level was significantly down-regulated at 36 h in the gills of mussels by siRNAs as compared with that in the control groups (Figures 6A,D). After *HcToll4* or *HcToll5* was knocked down, *HcThe* (Figures 6B,E), and *HcWAP* (Figures 6C,F) mRNAs were significantly down-regulated as compared with those without RNAi treatments, suggesting that *HcToll4* and *HcToll5* might be involved in regulating *AMP* (*HcThe* and *HcWAP*) expression.

## Discussion

As a significant class of proteins involved in the innate immunity of vertebrates, TLRs could provide the first step of defense against pathogens by recognizing PAMPs (Medzhitov, 2001). In invertebrates, Tolls are also involved in innate immunity by binding the ligand of Spätzle (Valanne et al., 2011). Many Toll genes were obtained from a de novo transcriptomic library of *H. cumingii*. In our previous study, we cloned and characterized five *HcToll* receptors, including *HcToll1, HcToll-I, HcToll2-1, HcToll2-2*, and *HcToll3* (Ren et al., 2013, 2014; Cao et al., 2016; Zhang et al., 2017). These *HcTolls* participate in *H. cumingii* innate immunity. Two other Toll (*HcToll4, HcToll5*) genes were identified in *H. cumingii* to study the diversity of Tolls in freshwater mussels. Compared with previously identified Tolls in *H. cumingii*, HcToll4 and HcToll5 have different domain structures. No LRR_CT domain could be found in HcToll4, whereas HcToll5 has only 1 LRR_CT domain. LRR_CT domain free Toll was rarely reported. In *H. cumingii*, HcToll3 also has no LRR_CT domain (Zhang et al., 2017). HcToll1 and HcToll2 both have two LRR_CT domains (Ren et al., 2013, 2014). Similar to HcToll3, HcToll4, and HcToll5 both have a LRR_NT domain located behind signal peptide (Zhang et al., 2017). From the phylogenetic tree, HcToll4 and HcToll5 showed closer relationship with HcToll3 than HcToll1, HcToll2s, and HcToll-I. TIR domain is an intracellular signaling domain found in IL-1 receptors, Toll receptors, and MyD88s and mediates the interactions between Tolls/TLRs and signal-transduction components. When activated, TIR domains recruit MyD88 and Toll interacting protein (Mitcham et al., 1996; Kawai and Akira, 2010). Similar to most found invertebrate Tolls, HcToll4, and HcToll5 both have one TIR domain located at the C-terminal of Tolls. In our previous research, we found a Toll with tandem TIR domains in the C-terminal (Ren et al., 2014; Cao et al., 2016). The genome of Pacific oyster contains two distinct loci encoding Tolls with 2 TIR domains; these Tolls were named as twin-TIR Tolls (Gerdol et al., 2017). Multiple Tolls found in *H. cumingii* suggested that the expansion and diversification of Tolls in *H. cumingii* genome.

Studying the expression level of functional genes in different tissues is useful to further understand their functions. Both *HcToll4* and *HcToll5* mRNA transcripts were most abundant in hepatopancreas. The hepatopancreas, which function for the fat body of insects and liver of mammals, are involved in humoral immune responses of invertebrates (Gross et al., 2001). The abundance of *HcToll4* and *HcToll5* in hepatopancreas may suggest their roles in the innate immune defense of *H. cumingii. HcToll2* also showed high expression levels in hepatopancreas (Ren et al., 2014) but no expression in the hemocytes of *H. cumingii*. By contrast, the *Toll* genes from *C. farreri* (*CfToll*) and *C. gigas* (*CgToll*) are mainly expressed in hemocytes (Qiu et al., 2007; Zhang et al., 2011).

In molluscas, hepatopancreas and gills are often used as target tissues to investigate the function of immune-related genes. In this study, hepatopancreas and gills were selected as our target tissues to investigate the expression pattern of *HcToll4* and *HcToll5* in *H. cumingii* presented with four different kinds of immune challenges. *HcToll4* and *HcToll5* transcripts showed different expression patterns with different immune challenges. In the gills or hepatopancreas with bacterial challenge, *HcToll4* or *HcToll5* was upregulated at one or more time points. However, *HcToll4* in hepatopancreas was not upregulated at any time point of virus or virus analogs challenge and was down-regulated at 24 h virus or virus analog challenge. *CfToll* transcript was up-regulated 6 h after LPS challenge (Qiu et al., 2007). *CgToll* was induced by *Vibrio splendidus* challenge (Zhang et al., 2011). In hemocytes, *C. nobilis CnTLR1* was induced by *V. parahaemolyticus*, LPS and Poly I:C challenges (Lu et al., 2016).

*Vibrio* is the most common bacterial pathogen in the mussel farming industry and is also used in immune challenge experiments (Tubiash et al., 1970). *Vibrio* injection could up-regulate the transcriptions of two *AMP* genes (*HcThe* and *HcWAP*). RNAi of *HcToll4* or *HcToll5* showed that the expression of *HcThe* and *HcWAP* was significantly inhibited. AMPs are one of the most important humoral factors that afford resistance to pathogen infection. Theromacin from *H. cumingii* possessed anti-bacterial feature, and its gene expression was up-regulated by Gram-positive and Gram-negative bacteria (Xu et al., 2010). The WAP domain has diverse functions, including proteinase inhibition (Sallenave, 2000) and anti-microbial activity (Wiedow et al., 1998). Crustin is a cationic cysteine-rich AMP that contains a single WAP domain at the carboxyl terminus and acts against microbial pathogens via both anti-bacterial and agglutinative activities (Arockiaraj et al., 2013). Invertebrate WAP proteins are critical in the host immune response against microbial invasion (Li et al., 2013). Therefore, *HcToll4* and *HcToll5* might have important roles in anti-vibrio immune defense by regulating the expression of *AMP* genes.

## Ethics statement

We declare that appropriate ethical approval and licenses were obtained during our research.

## Author contributions

YH and QR: designed the experiments; YH, KH, and QR: carried out the experiments; YH and QR: wrote the manuscript. All authors gave final approval for publication.

### Conflict of interest statement

The authors declare that the research was conducted in the absence of any commercial or financial relationships that could be construed as a potential conflict of interest. The reviewer SA and handling Editor declared their shared affiliation.
