# Supplementary material for: Cloning and Analysis of Gene Expression of Two Toll Receptors in Freshwater Pearl Mussel Hyriopsis cumingii
Source: Front Physiol. 2018 Mar 5;9:133. doi: 10.3389/fphys.2018.00133 (PMC5845394; doi:10.3389/fphys.2018.00133)
Supplement: Supplementary file 1 [file DataSheet1.docx]

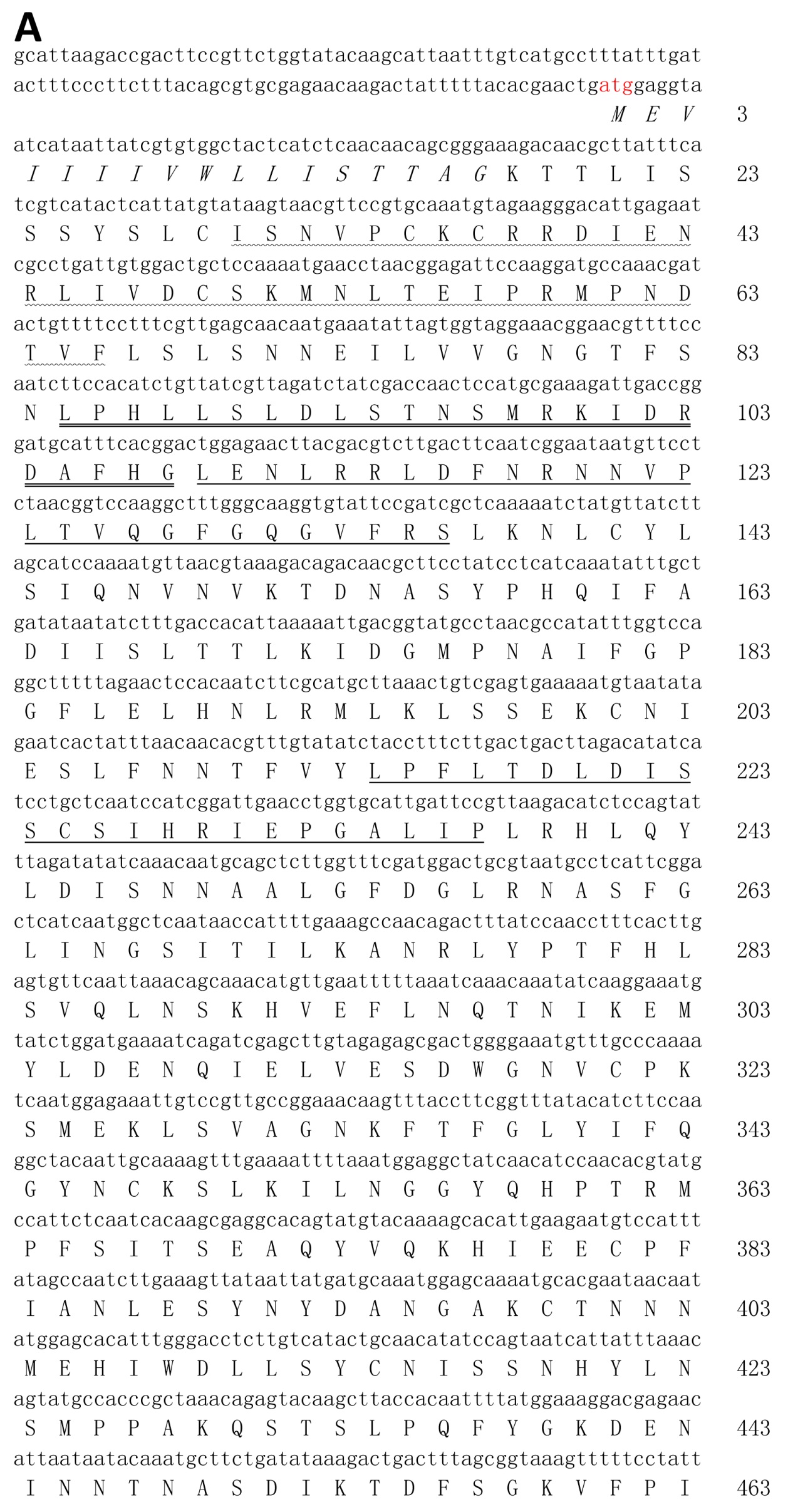


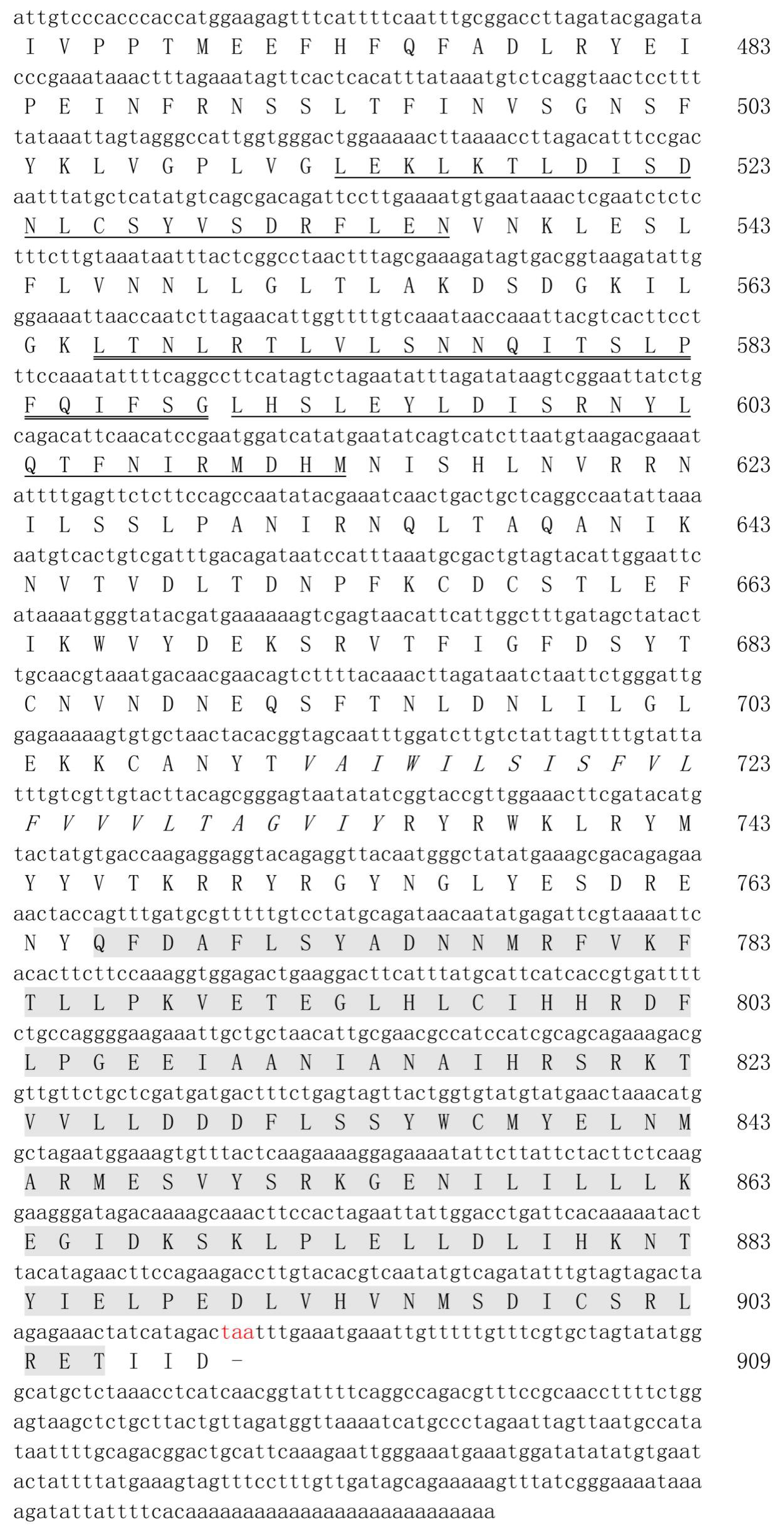


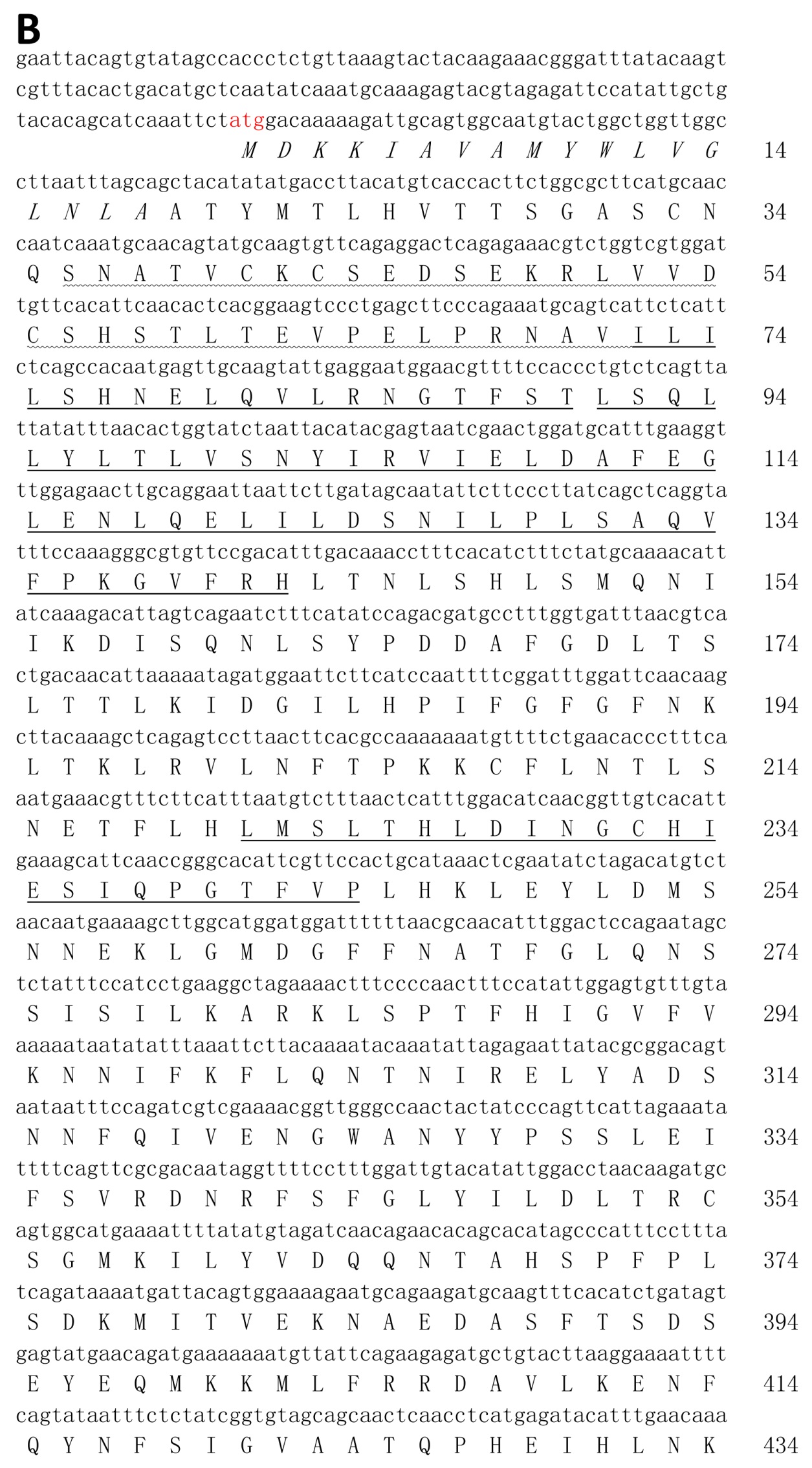


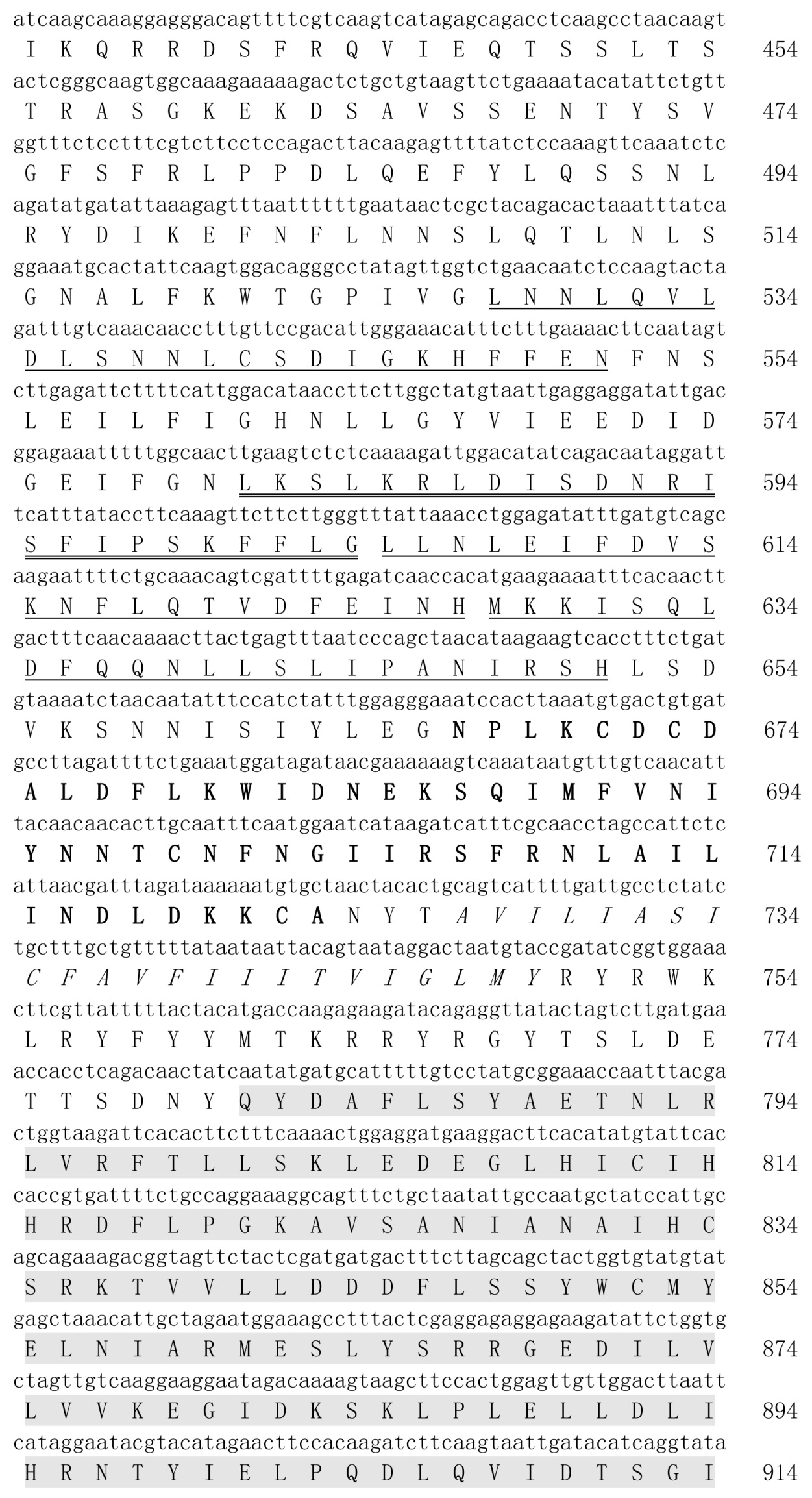


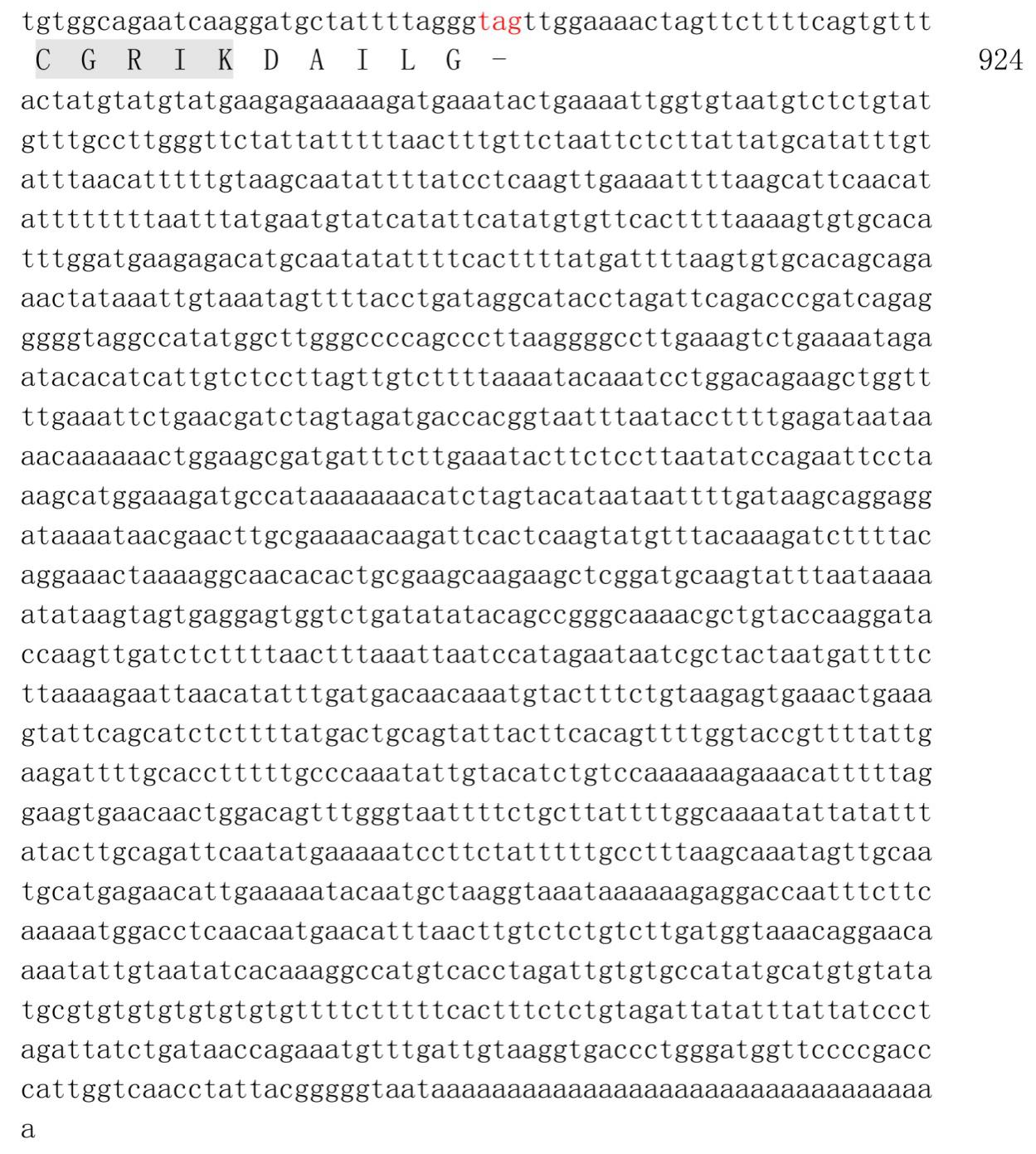


**Fig. S1.** Nucleotides and deduced amino acid sequences of HcToll4 (A) and HcToll5 (B) from *H. cumingii*. Signal peptides are labeled in italics. The wavy lines represent the LRR NT domains. The LRR domains are underlined, and the LRR TYP domains are double underlined. The LRR CT regions are shown in bold, and shaded sequences denote the TIR domains.


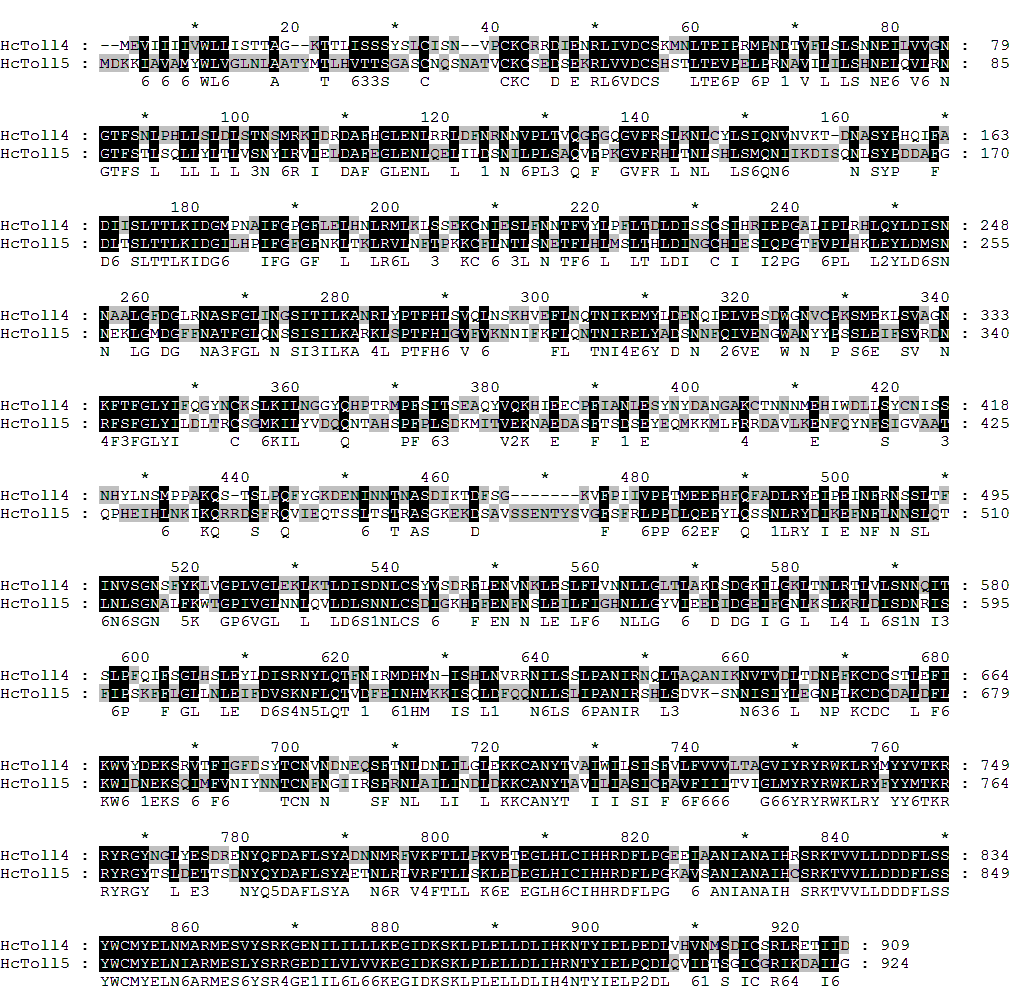


**Fig. S2.** Multiple sequence alignment by GENEDOC among HcToll4 and HcToll5 from *H. cumingii* according to the deduced amino acid sequence.
